# Trauma and Stem Cells: Biology and Potential Therapeutic Implications

**DOI:** 10.3390/ijms18030577

**Published:** 2017-03-07

**Authors:** Kabilan Thurairajah, Matthew L. Broadhead, Zsolt J. Balogh

**Affiliations:** 1 School of Medicine and Public Health, University of Newcastle, Callaghan, NSW 2308, Australia; thu.kabilan@gmail.com (K.T.); matthewbroadhead@me.com (M.L.B.); 2 Department of Traumatology, John Hunter Hospital, New Lambton Heights, NSW 2305, Australia

**Keywords:** trauma, stem cells, inflammation, DAMP, healing

## Abstract

Trauma may cause irreversible tissue damage and loss of function despite current best practice. Healing is dependent both on the nature of the injury and the intrinsic biological capacity of those tissues for healing. Preclinical research has highlighted stem cell therapy as a potential avenue for improving outcomes for injuries with poor healing capacity. Additionally, trauma activates the immune system and alters stem cell behaviour. This paper reviews the current literature on stem cells and its relevance to trauma care. Emphasis is placed on understanding how stem cells respond to trauma and pertinent mechanisms that can be utilised to promote tissue healing. Research involving notable difficulties in trauma care such as fracture non-union, cartilage damage and trauma induced inflammation is discussed further.

## 1. Introduction

Advances in modern trauma care in developed trauma systems achieved timely prehospital care, rapid diagnostics with simultaneous resuscitation and the focused evidence based management of individual injuries. A coordinated approach to these areas of care has led to improved mortality rates [1,2,3] and reduced preventable mortality [4]. Optimal recovery from major tissue injury relies on a patient’s intrinsic biology and regenerative capacity. Impaired biology may manifest as an inability to heal, suboptimal healing in the form of excessive scarring and trauma induced immune system dysfunction resulting in postinjury multiple organ failure. Ideal healing after trauma is a full return to preinjury condition without major scarring limiting function. Current research has focused on optimising the healing process through augmenting patient biology. Stem cell therapy is one potential avenue for achieving this goal. Stem cells are multipotent cells, capable of regenerating the body’s various tissues. This review aims to outline the basic biology of stem cells and their clinical potential in trauma care. Particular emphasis is placed on fracture healing, chondral healing and postinjury inflammation. To date, research has largely focussed on understanding stem cell behaviour and function though some translational applications are already reaching phase 1 clinical trials. There are many hurdles yet, before stem cell therapy reaches clinical practice. 

## 2. Stem Cell Biology

**Figure 1:**
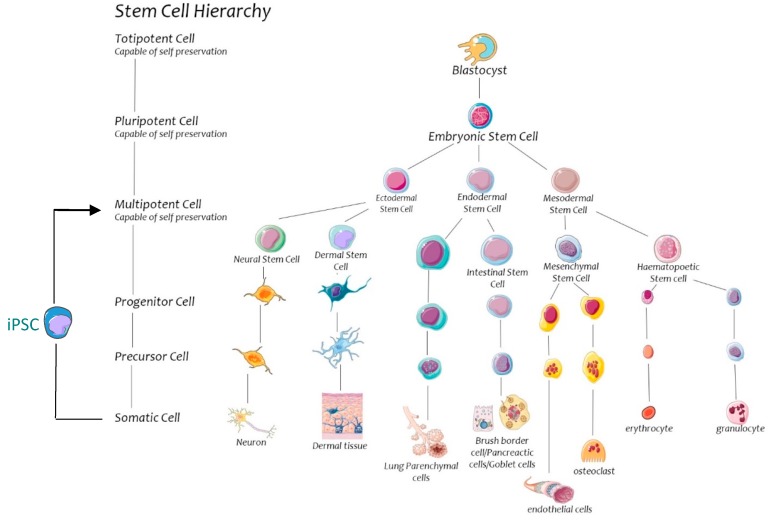
Totipotent cells of the blastocyst are capable of differentiation into embryonic and placental tissue. Stem cells can be grouped into three primary dermal layers (endodermal, ectodermal and mesodermal) and eventually mature into various somatic cells. Induced pluripotent stem cells (iPSC) are formed when somatic cells are manipulated to regress their maturity.

Regenerative cells in the body can be categorised by order of potency. The most potent cells are pluripotent blastocyst cells followed by multipotent stem cells, progenitor cells, and precursor cells [5] (Figure 1). These cells possess an inherent capacity to regenerate body tissues, however there are specific stem cells of interest with regards to trauma. Stem cells are undifferentiated cells that are capable of both self-renewal and differentiation into mature cells of various lineages. Stem cells develop from three primordial germ layers (endoderm, mesoderm and ectoderm). This review focusses on stem cells of particular interest in a trauma setting which include mesenchymal stem cells (MSC), haematopoietic stem cells (HSC), adipose derived stem cells (ADSC) and endothelial progenitor cells (EPC). Stem cells are found throughout the body in niches where a local microenvironment sustains their undifferentiated resting state [6,7]. Multiple mechanisms of molecular crosstalk exist between stem cells and neighbouring cells within their niches which control stem cell differentiation and self-preservation. Examples include Notch signalling and osteopontin regulation within endosteum [8]. The different classes of stem cells are found in characteristic niches; MSC and HSC are largely localised to the bone marrow, EPC to endothelium, ADSC to subcutaneous adipose tissue and satellite stem cells to muscle. It is worthy to note that there are new techniques for converting harvested somatic cells into induced pluripotent stem cells (iPSC) with multipotent regenerative potential. This allows for easy, less invasive harvesting of autologous stem cells regardless of patient age [9,10]. This process involves harvesting mature cells and inducing an escape from its terminally differentiated state via expression of genes typical of pluripotent cells. This “nuclear reprogramming” is possible through genetic manipulation such as nuclear transfer, cell fusion or transcription-factor transduction. This results in a breakaway from the natural cell cycle and induction of a pluripotent state, from which various tissue regeneration is possible [9,10]. Identification of surface markers is a mean of defining a stem cell population.

Mesenchymal stem cells (MSC) are multipotent stem cells capable of differentiation into any non-haematogenous cell along the mesodermal lineage such as osteocytes, chondrocytes, adipocytes and myelocytes. They are characterised by cell surface markers CD105, CD73, and CD90 [11,12,13]. MSC can be harvested from multiple sites including muscle, adipose tissue, bone marrow and the umbilical cord making autologous use possible. There are techniques for selecting, growing and expanding them in vitro in preparation for implantation in a host [14]. MSC are the most abundantly studied class of stem cell in terms of clinical trials. MSCs are poorly immunogenic as they lack the MHC class II molecule and its co-stimulatory molecules. They are also less likely to cause teratoma formation compared to pluripotent embryonic stem cells [14,15]. These qualities make them attractive for both autologous and allogeneic clinical use.

Haematopoietic stem cells (HSC) are capable of differentiation into myeloid or lymphoid cells. These cells are characterised by surface markers CD34, CD45, CD133 and Thy1 [16,17]. These are the cells used in allogeneic transplants for malignancies such as leukaemia, lymphoma and bone marrow failure. They are harvested from bone marrow and can also be found in peripheral blood [16,18]. 

Adipose derived stem cells (ADSC) are similar in potential to bone marrow derived MSC and demonstrate cell surface markers CD90, CD73 and CD44 [13,19,20] and may be harvested by means of lipo-aspiration under local anaesthesia [13]. 

Endothelial progenitor cells (EPC) have the potential for angiogenesis. They are present in the circulation and are identified by surface markers CD34, Flk-1 and Tie-2 [21].

## 3. Effects of Trauma on Stem Cells

Trauma causes structural damage to tissue, impairs tissue perfusion and triggers inflammation. The physiological response of stem cells to trauma include awakening from their resting state, mobilising from their niches, migrating towards sites of injury and differentiating to generate specific cells required for healing. Alternatively, pathological inflammatory response to injury can impair stem cell function and deplete stem cell population due to terminal differentiation, which leads to suboptimal tissue regeneration and poor outcomes [22,23,24].

**Figure 2:**
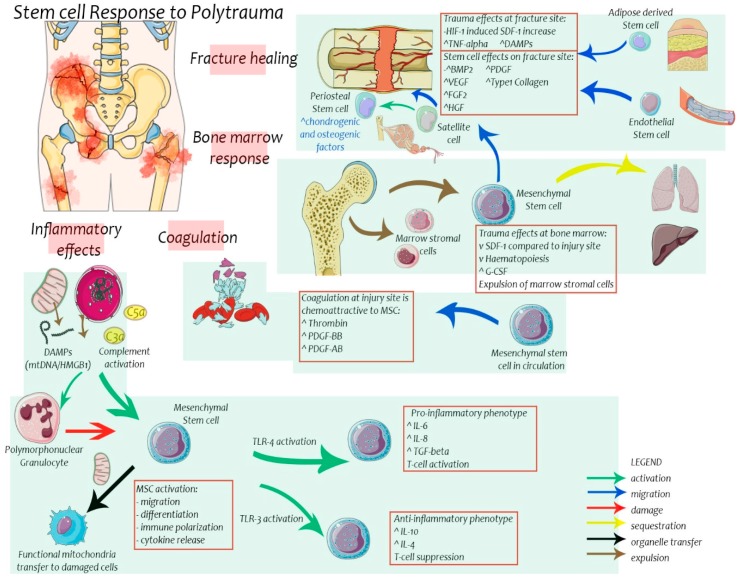
Various stem cell mechanisms are activated in response to severe injury. (1) Fracture healing involves multiple stem cells. Satellite cells play a vital role in activating Periosteal Stem Cells to release osteogenic and chondrogenic factors while also migrating to the fracture site to augment fracture healing directly. ADSC and ESC also migrate to the fracture site. (2) The SDF-1/CXCR4 axis facilitates migration of MSC away from bone marrow towards sites of injury. G-CSF (Granulocyte Colony Stimulating Factor) also favours release of stem cells from bone marrow. Severe injury is accompanied by bone marrow failure and expulsion of bone marrow stromal cells. Some of the circulating stem cells sequester in lung and liver parenchyma. (3) Coagulation occurs with the healing response to injury while severe trauma may result in coagulopathy. There is an increase in platelet factors and thrombin as a result, which is chemotactic to MSC. (4) Inflammation and immune activation follows injury and inadvertently involves stem cell function. DAMPs and complement proteins may activate and prime MSC while also stimulating Polymorphonuclear Granulocyte (PMN) to damage neighbouring MSCs. MSCs may be polarized into pro-inflammatory or anti-inflammatory phenotypes depending on the nature of Toll-like Receptor(TLR) activation. TLR-4 activation results in pro-inflammatory MSCs while TLR-3 activation gives rise to anti-inflammatory MSCs. MSCs also possess the ability to donate mitochondria to neighbouring damaged cells to improve cell survival.

Migration of MSC and HSC following trauma has been explained by numerous chemotactic interactions. Once such signal is the stromal derived factor-1/CXC chemokine receptor 4 (SDF-1/CXCR4) axis (Figure 2). This axis explains both retention of stem cells within niches and their migration towards sites of injury [18,25]. CXCR4 is a receptor on MSCs that binds SDF-1. SDF-1 is a protein that is physiologically expressed by bone marrow endothelial and stromal cells at concentrations higher than other tissues. Following injury, SDF-1 is produced at the site of tissue injury, at concentrations surplus to bone marrow, facilitating migration of MSCs away from bone marrow towards the site of injury. SDF-1 expression is regulated by hypoxia-inducible factor-1 (HIF-1) and nitric oxide (NO) [25]. Under normal physiological conditions, the SDF-1 concentration in bone marrow facilitates retention of MSCs. This has been validated in animal models of fracture and myocardial injury. Migration of MSCs towards SDF-1 has also been enhanced following therapeutic upregulation of CXCR4 [26,27].

Mechanical trauma initiates widespread cellular and humoral inflammatory response. Fu et al. [28] demonstrated in vitro that TNF-α is chemotactic for MSCs in a dose dependent manner. TNF-α acts on MSCs to induce intercellular adhesion molecule-1 (ICAM-1) expression making the cell more responsive to chemoattractant signal. TNF-α is released following trauma and is hypothesized to potentiate MSC migration towards sites of injury though this has not been studied in vivo. 

Ozaki et al. [29] investigated the chemotactic potential of multiple growth factors and cytokines using a microchemotaxis chamber to study MSC chemotaxis. Nine of the twenty-six cytokines studied demonstrated chemoattractant capacity. These factors showed capacity for both MSC migration and proliferation. Platelet derived growth factor-BB (PDGF-BB) exhibited the strongest single chemoattractant capacity, while combinations of two or more factors demonstrated an additive effect. Thrombin was also able to stimulate MSC migration. A limitation of this study was the use of cytokine concentrations greater than those found following trauma [30,31]. Nonetheless, there is an increased production of thrombin in trauma patients compared to healthy controls. This was observed by Dunbar et al in their assessment of trauma induced coagulopathy [32]. It remains to be investigated whether thrombin is a direct activator of MSC migration in trauma patients. 

Ritz et al. [33] studied the peripheral blood of 20 severely traumatised patients and demonstrated elevated numbers of CD34+ cells in circulation compared to control. Serial blood collections found progressively increasing numbers of CD34+ colony forming units (CFU) from admission until Day 7 compared to control. These CD34+ cells are believed to be haematopoietic cells or endothelial progenitors with pro-angiogenic capacity [33], demonstrating again a stem cell migratory response to trauma. 

Mechanical injury can be inhibitory and damaging to stem cells. Trauma induces activation of polymorphonucleated leucocytes (PMN) which have been shown to damage EPCs. Unlike PMN from controls, PMN from trauma patients caused EPC necrosis in vitro. This damage is thought to be mediated by increased reactive oxygen species (ROS) activity in trauma patient PMNs [34]. Two animal models have validated this mechanism [35,36]. Trauma also causes defects in bone marrow stromal growth. Bone marrow failure has been observed following major trauma affecting both myeloid and erythroid cell lines. There is an observed increase in circulating haematopoietic progenitor which coincides with anaemia due to the depletion of bone marrow stem cells. These patients also demonstrated a failure to respond to erythropoietin implying defective haematopoietic stem cell function [24]. 

The local microenvironment at a site of injury contains cytokines and growth factors that attract stem cells. In vitro migration assays on ADSC found that acute wound fluid was more chemoattractant than chronic wound fluid. Acute wound fluid has been shown to encourage ADSC proliferation while chronic wound fluid suppressed proliferation [37]. This suggests a growth factor or cytokine imbalance that impairs proper healing in chronic wounds. Injury also affects other stem cell function. Pathological changes in stem cell function have been implicated in development of multiple organ failure after trauma and post-traumatic osteoarthritis in joints. These are discussed in further detail below. 

## 4. Stem Cells in Bone Healing

Bone is capable of regeneration and remodelling following fracture. The AO Foundation (Arbeitsgemeinschaft für Osteosynthesefragen Foundation) have summarised the available scientific evidence in four basic principles for fracture treatment; fracture reduction, fracture fixation, the preservation of vascularity and finally early, safe mobilisation [38]. Of these principles, the preservation of vascularity and safe mobilisation following a fracture have been shown to have an impact on stem cell biology. Vascularity is important for the migration of stem cells to the site of injury, whilst mobilisation following injury provides a mechanical stimulus that promotes MSC differentiation. 

Fracture of a bone induces a systemic increase in the number of bone marrow MSCs [39]. While there are resident stem cells in bone marrow and periosteum, fracture causes the migration of stem cells to the site of injury [40]. There is a minimum number of stem cells required at the fracture site for union. Atrophic non-union is associated with a deficiency of MSCs at the fracture site [39,41].

Adequate vascularity is essential for stem cell migration from distant sites and survival [39,42]. Purified EPC delivery to rat fracture site resulted in increased angiogenesis and more rapid fracture union compared to controls [43,44]. The angiogenic effect of EPC at the fracture site was associated with increased local levels of pro-angiogenic factors hVEGF, hFGF2 and hHGF [44]. Similarly, transplanted MSCs are associated with increased callus volume and strength in mice fractures. These MSC were seen at the fracture site for up to 14 days following transplantation. The healing benefits were attributed to expression of bone morphogenetic protein-2 (BMP-2) by transplanted MSCs [41]. 

There are also techniques of augmenting the proangiogenic capacity of stem cells. Lin et al. [45] investigated the healing potential of modified ADSC on large segmental bone loss in rabbits. Engineered ADSCs were designed to express high and prolonged levels of BMP-2/VEGF by means of a viral vector and observed bony union and remodelling after 8 months. The bony defect was 10 millimetres in length. When unmodified ADSCs were transplanted to the fracture site non-union occurred. When modified ADSC were transplanted to the fracture site bony union occurred. Another stem cell vital to fracture healing is satellite stem cells from muscle tissue. These stem cells have been shown to play a part in fracture healing of both closed and open fractures by interacting with periosteum to stimulate callus formation as they are co-stimulated to repair damaged muscle fibres by trauma. This effect is achieved via release of osteogenic and chondrogenic factors and regulation of BMP dependent activation of periosteal bone progenitor cells [46]. While Abou-Khalil et al. [46] studied the contribution of satellite cells to bone healing in mice, they discovered a modifiable role for these cells in fracture healing. When satellite cells were inoculated directly into the fracture site, they differentiated into chondrogenic cells and greatly contributed to callus formation compared to controls without direct satellite cell inoculation to the fracture site. This may contribute to the available evidence based importance of preserving soft tissue coverage in managing fractures.

Stem cells have been shown to respond to mechanical stimuli. In vitro studies have demonstrated stem cell responses to stimuli such as tensile stress, compression, shearing, vibration and ultrasound. Osteogenic differentiation of stem cells has been demonstrated following tensile and compressive stress and ultrasound stimulation [47,48,49,50,51]. Reciprocally, Dai et al. [52] discovered that simulation of antigravity effects on rat MSC resulted in inhibition of osteoblastic differentiation. While some stem cells are stimulated by mechanical stimuli others show suppression. Low intensity pulsed ultrasound, which is an adjunct treatment for non-union, has been shown to promote osteogenic transformation of MSC while suppressing adipogenic transformation in vitro [50].

With increasing age the pool of osteoprogenitor cells available for osteogenic differentiation is reduced [53]. Stem cells from elderly humans have a preponderance for adipocytic differentiation as opposed to osteogenic [54]. Liposomal Wnt3a protein (L-Wnt3a) is responsible for regulating the differentiation of bone marrow stem cells towards adipocytic or osteogenic progenitors. Leucht et al. [55] discovered that aged mice had a reduced liposomal Wnt3a protein level compared to young mice which coincided with the finding of reduced osteogenic capacity and increased fatty change in the bone marrow. The incubation of bone graft with Wnt3a protein increased its osteogenic regeneration compared to control.

Human studies involving stem cell therapy for non-union have largely been case reports and non-randomised case control studies with one randomised control study published. Results have been encouraging though insufficient for routine clinical use [56]. Liebergall et al. [57] conducted a randomised control trial to determine the safety of using iliac crest MSC together with platelet rich plasma and liquid demineralised bone matrix in management of distal tibial fractures. They reported no adverse effects in the intervention group along with accelerated fracture union compared to control, six weeks versus 12 weeks, respectively. Kuroda et al. [58] published pilot data from a human trial utilising autologous CD34+ cells in an atelocollagen scaffold and iliac bone graft from patients with tibial or femoral non-union. CD34 therapy achieved a 71.4% bony union rate at 12 weeks compared to an 18.1% rate of union at 12 weeks in control patients. This study had a sample size of seven and did not standardise the interventions. Stem cell therapy was an addition to standard care after non-union was defined as failure of radiological union at nine months from injury with no progress of union in the three months before enrolment in the trial. The authors report 100% bony union at 36 weeks with all patients returning to work without ongoing pain.

## 5. Stem Cells in Chondral Healing

Stem cell function is associated with the recovery of articular cartilage injury and with the pathomechanism of post-traumatic osteoarthritis [59,60]. In response to injury, MSC within articular cartilage differentiate to produce fibroblastic cells instead of chondrogenic cells. This process is partly due to ADAMTS5 (a disintegrin-like and metallopeptidase with thrombospondin type 1 motif 5) protein mediated TGFβ-1 signalling. ADAMTS5 is a cartilage aggrecanase. Trauma induces an upregulation of ADAMTS5 synthesis in fibroblast cells. The presence of ADAMTS5 promotes MSC differentiation into fibroblasts while absence promotes differentiation into chondrocytes [60]. In mice with ADAMTS5^−/−^ deletion, there is an increased knee cartilage aggrecan content and less joint tissue fibrosis in response to trauma compared to controls [61,62]. This demonstrates a change in stem cell differentiation induced by trauma and correlates with post-injury osteoarthritis.

Articular cartilage can be generated by stem cells in vivo and in vitro. MSCs and ADSCs from various tissues have been utilised for this purpose. Animal models have demonstrated varying success with cartilage regeneration. Diekman et al. [63] and Mak et al. [64] compared the protective effects of MSC against post-traumatic osteoarthritis. Following joint trauma, mice received intra-articular injection of MSC isolated from Murphy Roths Large (MRL) mice. MRL mice are known as “superhealer” mice as they have a remarkable capability of cartilage regeneration. This was compared against MSC isolated from Black 6 (B6) mice which are naturally unable to regenerate cartilage after injury. Diekman et al. utilised a mouse model in which a tibial plateau fracture was caused by a 10N load. At eight weeks, mice that received intra-articular injection of MSC demonstrated better preservation of cartilage compared to control mice which did not receive MSCs. MSC from both MRL and B6 mice showed cartilage preservation [63]. This suggests that the inherent ability of MRL mice to regenerate chondral tissue is not due to suprapotent stem cells but suggests chondral regeneration from a more beneficial regulation of stem cell function. Mak et al. [64] also studied the effect of MSCs chondral healing. A needle was used to cause a bony defect in a mouse model, coring cartilage to a depth of 2.7 mm. At four weeks, there was no regeneration of cartilage but reduced cartilage proteoglycan breakdown was observed. The MSC isolated from MRL mice were observed to home into the site of injury better than MSC from B6 mice. It is difficult to compare the results of these two studies as different grading systems were used to analyse the cartilage histologically, the nature of the trauma was different and the outcomes were measured at different time points.

Intra-articular injection of stem cells has successfully improved healing of chondral defects while subcutaneous injection showed no effect on cartilage healing in murine models, citing the mode of delivery of exogenous stem cells as an important variable to consider [19,63]. It is imperative to keep in mind that ex vivo expansion of stem cells decreases their ability to home in to sites of injury [65]. This makes the mode of delivery important if the stem cells have not been modified to improve their migration to sites of injury [27]. Liu et al. [51] have developed a different approach to treating cartilage defects by generating chondral grafts from autologous infrapatellar fat pad stem cells in patients with osteoarthritis. The stem cells were cultured for six weeks with TGF-β3 and BMP-6 to generate sizeable chondral grafts measuring more than 2 cm in diameter.

Saw et al. [66] conducted a clinical trial on 50 patients with chondral injury. The patients aged 18 to 50 were randomised to receive autologous stem cells from blood and hyaluronic acid or control who received hyaluronic acid alone. Each patient underwent arthroscopic subchondral drilling and abrasion chondroplasty to the chondral defects, then a series of five, weekly knee injections and a subsequent arthroscopy at 18 months when a chondral biopsy was taken. Histological analysis and MRI examination revealed improved chondral regeneration in the stem cell group compared to control. No functional scores were measured however. A pilot study on cartilage healing and functional improvement in humans has yielded promising results, although patients were being treated for chronic chondral defects in the form of osteoarthritis rather than acute chondral injuries from trauma. Oroszco et al. [67] have utilised bone marrow derived MSCs to treat knee osteoarthritis in 50 patients with symptoms unresponsive to medical and physical therapy. MSCs were infused intra-articularly and patients were followed up for 12 months. Pain and functional scores were significantly improved along with MRI evidence of improved cartilage quality. Similarly, Vangsness et al. investigated the effect of intra-articular injection of allogenic MSC following arthroscopic partial meniscectomy. Fifty-five patients were enrolled in the double blind randomized control trial. MSC therapy resulted in meniscal tissue regeneration and improved pain scores in patients with concomitant osteoarthritis. No serious adverse events were reported at two-year follow-up [68].

MSC therapy promises to revolutionise the management of chondral injury be it osteoarthritis or chondral defects following trauma. Current efforts are focused on determining the optimum source of MSCs, ex vivo modification prior to implantation, route of administration, use of scaffolds and safety. Multiple reviews of available literature on MSC therapy for osteoarthritis have concluded that more research is required prior to clinical application [69,70,71]. Although MSC therapy for osteoarthritis and post-traumatic arthritis aim to regenerate hyaline cartilage, the microenvironment within these two pathologies differs. It is unclear whether MSC therapy for osteoarthritis will also effectively treat post-traumatic arthritis.

## 6. Stem Cells in Post-Injury Inflammation and Multiple Organ Failure

Postinjury MOF is considered as a result of dysfunctional inflammatory response to trauma. Stem cells possess immunomodulatory functions. MSC are capable of transformation into both pro-inflammatory and anti-inflammatory cells dependent upon secreted mediators in the postinjury inflammatory response. Toll-like receptors (TLR) have a role in regulating MSC inflammatory polarity [72,73,74]. TLR are surface receptors that bind pathogen molecules and damage associated molecular patterns (DAMPs). DAMPs are endogenous products of cell destruction following trauma that are also capable of immune cell activation. Examples include mitochondrial DNA (mtDNA), High-Mobility Group Box-1 protein and S100 proteins.

MSC have been shown to express surface TLR 1-9 depending on their maturity [73]. For example, TLR-9 expression is lost as MSC mature into osteoblasts. TLR-9 activation was found to increase migration of MSC, partially through increased matrix metalloproteinase 13 (MMP-13) production [75]. TLR-9 functions to detect CpG-motifs (cytosine-guanine oligodeoxynucleotide with phosphodiester link) which are abundant on microbes. Interestingly, similar CpG-motifs are found in human mtDNA (a potent DAMP) which is liberated into extracellular spaces following trauma [75,76]. When DAMPs are released after trauma, they bind TLR on MSC and affect the immune response to injury. TLR-4 activation on MSC results in pro-inflammatory mediator release while TLR-3 activation results in anti-inflammatory mediator release. When these activated MSCs were co-cultured with human monocytes and lymphocytes from circulation the TLR-4 primed MSC culture activated T-lymphocytes while TLR-3 primed MSC culture suppressed T-lymphocytes [72]. These immunomodulatory effects have also been shown in sepsis. Nemeth et al. [77] demonstrated an immunosuppressive effect of MSC in a murine sepsis model. MSC therapy following induction of sepsis was found to result in increased anti-inflammatory IL-10 secretion from lung macrophages. This immunosuppressive effect was attributed to MSC secretion of prostaglandin E2. There was a resultant observation of reduced neutrophil migration and oxidative damage. Beyond that, MSC have demonstrated another ability to help fight infection. Observations by Islam et al. [78] found exogenous MSC actively donating mitochondria to neighbouring host lung epithelial cells in a murine model of acute lung injury. The mitochondria were packaged in microvescicles that were incorporated into epithelial cells. This resulted in more ATP generation within epithelial cells, more surfactant secretion and improved survival compared to control animals which received MSC with defective mitochondria. 

Trauma induced activation of PMN and macrophages results in damage to stem cells. PMN along with stem cells migrate to sites of injury however they largely migrate at different timepoints following trauma. PMN are activated to release ROS that inadvertently damage surrounding cells including the recruited stem cells. If the injury is severe enough and PMN activity persists (frequently due to delayed apoptosis) when stem cells are recruited, the stem cells can be injured [34,35,36]. The acute inflammatory phase following trauma has also been shown to affect the efficacy of exogenous stem cell implantation in a rat model of traumatic brain injury. Macrophage activation by trauma was found to result in direct phagocytosis of embryonic stem cells that were administered after traumatic brain injury in rats. Following this study, Molcanyi et al. [22] concluded that trauma resulted in priming of macrophages against otherwise immunologically privileged stem cells.

Luo et al. [23] have demonstrated a correlation between the incidence of multiple organ failure (MOF) after trauma and a decline in number and function of circulating EPC. Using a pig injury model there was an increase in EPC migration and adhesiveness following trauma with subsequent decreases in these phenomena as MOF developed. There was an observed decline in migratory and cell adhesive function of the EPC that preceded the decline in number of circulating EPC. Hence, a potential pathogenic correlation between declining EPC function and development of MOF exists. In a subsequent study, Tianhang et al. [79] demonstrated protective effects of transplanted EPC in reducing the incidence of MOF in the pig injury model. Pigs that received EPC injection after trauma showed histological evidence of increased angiogenesis in vital organs compared to control. This correlated with a lower incidence of MOF in the treatment group compared to control.

Bone marrow failure is a part of multiple organ failure after severe trauma. Livingston et al. [24] discovered that critically injured patients in ICU required weekly blood transfusions despite no obvious ongoing blood loss. These patients were anaemic with minimal elevation of reticulocyte count despite markedly elevated erythropoietin concentrations in plasma. They demonstrated a marked increase in circulating bone marrow progenitor cells compared to control but showed impaired bone marrow synthetic function. Iliac crest bone marrow aspirates from these severely injured patients showed greatly impaired stromal growth compared to healthy controls. Similarly, Cook et al. [80] also found elevated bone marrow progenitor cells in their study of 83 severely injured patients. In addition, G-CSF (Granulocyte Colony-Stimulating Factor) was markedly elevated compared to control, even more so in patients with shock. A positive correlation was identified among G-CSF concentration, anaemia, transfusion requirements and infective complications. This phenomenon may be explained by the discovery of Petit et al. [81] who found that G-CSF causes a reduction in bone marrow SDF-1 protein. G-CSF stimulates bone marrow neutrophil elastase release which degrades SDF-1 locally thus leading to bone marrow progenitor cell egress by virtue of the SDF-1/CXCR4 axis. This was validated in a murine model. This leaves us with evidence that the hyper-inflammatory state and elevated G-CSF concentrations following severe trauma result in impairment of bone marrow stem cell haematopoiesis and susceptibility to infection.

An animal model has shown reversal of bone marrow failure after injury by virtue of MSC therapy. Gore et al. [82,83] devised two mouse models to investigate the therapeutic effects of allogenic MSC on bone marrow failure after injury. The injury was in the form of lung contusion and haemorrhagic shock or chronic stress by virtue of restraining the mice daily over seven days. MSC therapy was administered with resuscitation and outcome measures included plasma G-CSF concentration, bone marrow cellularity, bone marrow growth potency and number of circulating bone marrow progenitor cells. MSC therapy was shown to successfully return bone marrow cell counts and function to that of healthy control and normalise plasma G-CSF concentration. There was no difference in the observed number of circulating bone marrow progenitor cells.

Acute respiratory distress syndrome is encountered in severe trauma and there is an emerging role for MSC in treating this syndrome. Hayes et al. [84] investigated the effects of human MSC administration in a rat model of ventilator induced lung injury. The MSC were administered intravenously and gave rise to improvement in lung compliance, better restoration of lung parenchymal damage, reduction of inflammatory mediator release and reduction in alveolar inflammation compared to control animals. Maron-Gutierrez et al. [85] also looked at how human MSCs affected lung injury in mice following lipopolysaccharide (LPS) induced lung injury. The MSCs were administered a day after LPS and reduced inflammatory changes and atelectasis compared to control mice while also modulating macrophage phenotype towards anti-inflammatory function. Interestingly, MSCs, when administered subcutaneously or intravenously, tend, initially, to sequester in pulmonary circulation [86,87]. This has been attributed to their large size and can give rise to embolic events and raise pulmonary artery wedge pressure [87]. Wilson et al. [88] conducted a phase I clinical trial to determine the safety of intravenous allogeneic MSC therapy in nine patients with moderate to severe ARDS. No infusion related adverse effects were reported and a subsequent phase II study has commenced. A mortality rate of 22% was reported to be similar to documented mortality rates for moderate to severe ARDS. 

## 7. Stem Cells in Wound Healing

Skin wounds heal through phases of inflammation, proliferation and remodelling [89]. Stem cells are physiologically involved during these phases of healing with animal studies and small sample clinical studies showing accelerated wound closure without scar formation following MSC therapy [90]. Resident cutaneous interfollicular stem cells are derivatives of mesenchymal stem cells and collectively they contribute to immunomodulation, angiogenesis, chemotaxis, anti-fibrosis and preservation of stem cell homeostasis [90,91].

Skin trauma causes the local and systemic release of IL-6, TNF-α, and IL-1β. Bader et al. [92] investigated the effects of these cytokines on stem cell proliferation. IL-6, TNF-α and IL-1β inhibited stem cell proliferation when cultured individually. This effect was observed to be reversed in the presence of erythropoietin. Erythropoietin, which is physiologically present in skin, when cultured together with IL-6 and TNF-α, stimulated stem cell proliferation. This prompted more research into investigating the ability of topical erythropoietinas an activator or resident stem cells.

Niyaz et al. [93] studied MSC application to rat traumatic necrotic skin flaps. MSC therapy improved healing and reduced the skin defect compared to control. Likewise, Wu et al. [94] demonstrated MSC therapy enhanced the rate of tissue healing and angiogenesis in diabetic and non-diabetic rats compared to control. Besides MSC, ADSC has also been utilised in animal models of traumatic wounds. Kim et al. [95] found improved wound healing in rats following ADSC therapy. They attributed the healing benefits to up-regulation of Type 1 collagen production, increased mRNA expression of extracellular matrix proteins and increased dermal fibroblast migration. ADSCs were also found to secrete growth factors like insulin-like growth factor (IGF), platelet derived growth factor (PDGF) and KGF. ADSC therapy resulted in quicker re-epithelialisation and reduced wound size compared to control.

MSCs have also been incorporated into skin substitutes that aim to promote wound healing. Scaffolds represent skin substitutes that serve as an extracellular matrix (ECM) for cell migration, proliferation and revascularisation [96]. Nie et al. [97] used human cadaveric skin with preserved ECM structure as a scaffold for ADSC, growth factors and cytokines. The scaffolds were processed to remove antigenic features. This was used in diabetic rats and resulted in improved healing through enhanced neovascularization, granulation and re-epithelialisation. Similarly, Formigli et al. [98] used bovine tendon as a scaffold for MSC in treatment of skin defects in rats and observed enhanced healing.

## 8. Stem Cells in Muscle Healing

Muscle tissue have resident stem cells in the form of satellite cells [99]. Satellite cells comprise a heterogenous group of muscle stem cells and progenitor cells [100]. Although a very vascular tissue, muscle tissue is largely incapable of complete regeneration following injury. Following trauma, be it laceration, contusion or strain, muscle often heals with fibrosis which results in impairment of contractility and function [101]. Fibrosis following trauma is attributed to a rise in TGF-β1 which stimulates satellite cell differentiation into myofibroblasts. Thus, increased Type 1 collagen is deposited and scarring begins [102,103]. Physiologically, satellite cells aid in recovery from muscle injury along with MSC that migrate from other tissues. Muscle injury induces satellite cell activation to give rise to myoblasts which ultimately differentiate into muscle fibers [100]. Satellite cells are robust in neonates but conversely quiescent in adults. The reasons for this drastic difference in their reactivity is unclear [104]. Thus, research in stem cell therapy for muscle regeneration should focus on both activation of resident satellite cells and exogenous MSC therapy.

Skeletal muscle injury in the form of eccentric contraction is sufficient to stimulate mobilisation of MSC into circulation. Ramirez et al. [105] found a surge in circulating MSCs in healthy volunteers following a 21 km race. The muscle injury was validated using a creatine kinase assay before and after the race. Cardiac muscle injury was assessed and not encountered in any of the volunteers by virtue of Troponin I assay. Satellite cells normally reside in a resting state on the basal lamina of myofiber sarcolemma. Trauma causes disruption of this niche and results in activation of satellite cells to migrate, divide and differentiate [106,107].

Von Roth et al. [108] studied the impact of MSC therapy on healing following muscle crush injury in rats. The soleus muscle was crushed using artery forceps and autologous mesenchymal stem cells, harvested from tibial biopsy, were injected into the muscle a week later. They measured significant improvements in muscle contractility following MSC therapy compared to control. Histological analysis also demonstrated less fibrosis. Winkler et al. [109] utilised the same model to demonstrate significant benefit in contractility from MSC therapy immediately following trauma and one week later.

## 9. Stem Cells in Angiogenesis

Vascularity is critical to healing. Trauma may result in acute and delayed injury to capillaries in the form of direct injury, occlusion from clot or raised interstitial pressure and iatrogenic injury during surgical management of trauma. Stem cells have direct and indirect effects on angiogenesis following injury. Some studies have demonstrated ESC migration to sites of injury and subsequent direct involvement in neovascularization [21,110,111,112]. Other studies have demonstrated an indirect stimulation of angiogenesis by ESC via secretion of growth factors, cytokines and microvesicles that stimulate resident endothelial cells to facilitate angiogenesis [113,114,115]. The proangiogenic property of ESC is also shared by MSC and ADSC [94,116,117,118]. The proangiogenic function of these stem cells ultimately contributes to improved fracture healing [43,44], capillary healing [118], wound healing [94] and reduced inflammatory complications [79]. 

## 10. Current State of Stem Cell Research

Stem cell therapy is gathering impetus in translational research. At present, there is still a paucity of published clinical trials despite numerous commenced and completed phase 1, 2 and 3 trials in clinical trial registries. With regards to stem cell therapy in fracture healing, the highest level of publication yielded from our literature search was two level II studies: one published level II clinical trial [57], and one published level II pilot study [58]. There are numerous animal studies and basic science papers. 

Studies on chondral healing included one level II clinical trial [66] which did not evaluate functional outcomes of therapy, two level II studies looking at functional outcome however in patients with osteoarthritis not acute trauma [67,68] and numerous animal studies and basic science papers. There is an abundance of papers and clinical trials on stem cells and wound healing. At present the Wound Healing Society guidelines state the role of stem cells in current practice is promising yet still undefined [119]. Most published clinical trials are regarding stem cell use in critical limb ischaemia. A few good reviews of wound healing properties of stem cells are available and all conclude that more research is required prior to translation into clinical practice [90,120,121,122]. Stem cell research on inflammation after trauma has yielded two Level III studies [24,80] and multiple animal studies and basic science papers. Similarly for stem cells in muscle healing there is one Level III study [105] and numerous animal studies and basic science papers.

## 11. Future Directions

While stem cell therapy holds much promise for improving patient outcomes following trauma through tissue regeneration and immunomodulation, current literature shows that outcomes are inconsistent. There are yet many challenges to overcome before stem cell therapy is clinically applicable. Further research is required in the form of animal models and clinical trials to characterise the mechanism of action and effects of stem cells on tissue repair. Additionally, whether these potential therapies result in functional improvement and side effects remains to be seen.

Multipotent cells are immature and close regulation of their differentiation and tissue regenerative potential is essential to prevent teratoma formation and malignant transformation. Only one paper in this review reported dysplastic complications of stem cell therapy, however this represents a very serious morbidity. This reinforces the importance of thoroughly understanding stem cell mechanisms and the involved regulatory factors. Kuroda et al. [58] performed a human trial of CD34+ implantation for fracture non-union. Adverse outcomes included uterine cervical dysplasia, splenomegaly, liver enzyme derangement and deep venous thrombosis. In this trial, all patients studied had at least mild adverse effects. These problems should be assessed with phase I, II and III trials. 

However, some modalities of stem cell therapy utilise ex vivo regeneration of tissue and subsequent implantation of these grafts. This may be in the form of cartilage, skin or ligaments. Tumorigenesis may not be as large a concern here, however, more research is required to understand how the host responds to these grafts and how the grafts respond to physiological stresses such as weight bearing. Another challenge with stem cell therapy is determining the most effective mode of delivery of stem cells. Gao et al. [86] observed that systemically delivered MSC largely sequestered in lung and liver parenchyma. This implies a reduced number of MSCs that successfully seed into sites of injury, reducing treatment efficacy. There may also be embolic complications and strain on cardiac function [87]. It is unclear whether these adverse effects are concentration dependent or whether they can be rectified with direct inoculation of MSC into sites of injury. Optimum MSC delivery is likely to differ with the nature of the pathology being treated. Furthermore, results from clinical trials on MSC therapy for specific pathologies are not likely transferable to other pathologies. This is due to the complexity of these pathologies and MSC function which is not yet fully understood.

There are still many hurdles to overcome before stem cell therapy can become a part of clinical practice. Some applications are closer to clinical applicability than others. Current concepts and techniques are striving to manipulate the native stem cell population, and utilise autologous cultured stem cells and allogeneic stem cells with or without scaffolds. As methods of harvesting, processing and delivering stem cells improve, we edge closer to new modalities in the management of trauma and healing.
